# Sudden Collapse in the First Trimester: Report of Hyperacute Renal Failure Secondary to Collapsing Glomerulopathy as the Initial Presentation of Lupus

**DOI:** 10.7759/cureus.1509

**Published:** 2017-07-24

**Authors:** Pooja Sethi, Jennifer Treece, Chidinma Onweni

**Affiliations:** 1 Department of Cardiology, Quillen College of Medicine, East Tennessee State University; 2 Internal Medicine, Quillen College of Medicine, East Tennessee State University

**Keywords:** hyperacute renal failure, lupus nephritis, collapsing glomerulopathy, systemic lupus erythematosus (sle), pregnancy

## Abstract

Hyperacute renal failure is rarely the initial presentation of systemic lupus erythematosus (SLE). Pregnancy can predispose untreated lupus nephritis to acute renal failure. Collapsing glomerulopathy (CG) type of renal failure is not a new clinicopathological entity. There have been documented cases prior to 1979. It is thought that detection bias coupled with the predilection for HIV has caused this form of glomerulopathy to be incorrectly named or diagnosed as 'malignant focal segmental glomerulosclerosis (FSGS)'. This is a case of CG described in lupus nephritis. We present a case of untreated lupus in a female in whom pregnancy triggered the exacerbation of lupus nephritis that presented as collapsing glomerulopathy.

## Introduction

Systemic lupus erythematosus (SLE) can affect the entire body causing multiple organ manifestations. Although nephritis in SLE is a well-known entity, hyperacute renal failure as the initial presentation of SLE is uncommon. Pregnancy can predispose a patient with untreated lupus nephritis to develop hyperacute renal failure. Collapsing glomerulopathy (CG) is a relatively new clinicopathological entity described with lupus nephritis. This can lead to rapid progression of renal failure to end-stage renal failure and often requires dialysis [1-2]. We present the case of a patient with undiagnosed and therefore untreated SLE that developed hyperacute renal failure in the form of CG when she became pregnant. Her initial presentation for SLE was rapidly progressive acute renal failure.

## Case presentation

A 19-year-old six-week pregnant African American female presented to the emergency department (ED) with a two-day history of progressive shortness of breath. She had severe orthopnea for the previous two weeks requiring her to sleep propped up on four pillows. She had multiple ED visits over the last two years with hypertension and documented 15mg/dL proteinuria (on urinalysis) prior to this hospital admission. She had been referred to an outpatient nephrologist previously but did not keep the appointment. On physical examination, her blood pressure was 173/109, heart rate was 105 beats per minute, respiratory rate was 22 breaths per minute, and oxygen saturation was 94% on room air. On respiratory exam, her lungs had diffuse rales bilaterally. The cardiovascular and abdominal exams were benign. Her extremities had bilateral pedal edema. Her labs showed normal white count, hemoglobin 7gm/dL, hematocrit 22.5%, platelets 133, creatinine 16.7mg/dL (0.4 mg/dL two months prior), and blood urea nitrogen (BUN) 92. Urinalysis showed greater than 30mg/dL proteinuria. She received emergent hemodialysis. Further work up for her acute renal failure revealed positive antinuclear antibodies (ANA) titer of 1:640, and her anti-double-stranded DNA (anti-dsDNA) antibodies were 1204 IU/mL (markedly elevated. ≤4IU/mL negative, 5-9 IU/mL indeterminate, ≥10 IU/mL Positive).

She was negative when screened with fourth generation combined enzyme-linked immunosorbent assay antibodies/ p24 antigens for human immunodeficiency virus (HIV). She denied having a history of intravenous drug use. Given her anemia, renal failure, elevated ANA, and positive anti- dsDNA antibody, the patient satisfied the American College of Rheumatology criteria for SLE [3]. Her renal ultrasound revealed diffusely increased echogenicity and renal biopsy showed 80% of the glomeruli were globally sclerosed with one visualized glomerulus showing collapsing basement membrane, confirming the diagnosis of CG. There was diffuse interstitial fibrosis and vasculopathy of the kidneys as well. She was discharged on hemodialysis until improvement of her renal failure. Since hospital discharge, the patient’s renal function has improved, and she no longer requires hemodialysis at this point in time.

## Discussion

Epidemiology

The prognosis for CG is poor [4-6]. CG is classified as a variant of focal segmental glomerulosclerosis (FSGS) [6]. The mortality of patients with SLE is three times higher than the general public [7].

Risk Factors

CG is a new clinicopathological entity described in patients with SLE presenting with rapidly progressive renal failure. Although newly recognized to be associated with SLE and other collagen vascular disease, CG is known to be associated with other autoimmune diseases, HIV, cytomegalovirus (CMV), tuberculosis, parvovirus B19, hepatitis C virus, and induced by medications (i.e., pamidronate, valproic acid, interferon-alpha, or bisphosphonates) [1,4,6-8]. CG is most commonly seen in African American women with a history of intravenous drug use [6]. Pregnancy is an additional risk factor for CG as pregnancy can predispose a patient with untreated lupus nephritis to develop hyperacute renal failure in the form of CG [9].

Clinical Features

The patient, in this case, had anemia, renal failure, elevated ANA, and positive anti- dsDNA antibody, so the patient satisfied the American College of Rheumatology criteria for SLE [3]. CG is found in patients with SLE who present with acutely progressive renal failure, and patients with CG have signs and symptoms of renal failure including proteinuria, which is often in the nephrotic range [7], as well as elevated creatinine and BUN, low glomerular filtration rate (GFR), and fluid overload that manifests as dyspnea, orthopnea, bibasilar rales, and bilateral lower extremity edema. Like the patient presented in this case, a large percentage of patients with CG progress to end-stage renal disease [6]. Although pre-eclampsia, eclampsia, and sepsis are the most common causes of pregnancy-related acute renal failure [10], CG secondary to untreated lupus nephritis in a pregnant patient is a rare cause of pregnancy-related acute renal failure.

Diagnosis

Renal biopsy is required to visualize the collapse of the glomeruli to confirm the diagnosis of CG. CG is diagnosed by inspecting renal tissue samples under light microscopy, which display pseudo-crescents from the collapse of glomerular capillaries with proliferation and swelling of the overlying podocytes [6]. Under electron microscopy, CG appears as a collapsed glomerular basement membrane with foot process effacement and hypertrophy of the overlying podocytes [6].

Treatment

Treatment of the underlying cause of CG, whether infectious in nature or secondary to an autoimmune disorder like SLE, is important to controlling the progression of the disease. If CG is thought to be medication-induced, cessation of the offending medication is vital for recovery. Addressing the underlying cause of the CG leads to improvement in renal function [6]. Hemodialysis is often required initially when patients present with CG but may be eventually discontinued if the underlying cause of the CG is treated [6]. There is not a specific treatment for CG other than treatment of the underlying cause. CG may respond to treatment with immunosuppressive therapy and steroids, such as mycophenolate mofetil or cyclosporine and prednisone, respectively [2,5]. Intravenous immune globulin (Ig) has also been used to treat CG [8]. Other than treating the underlying cause, the optimal treatment for CG is not known. The treatment also includes controlling the patient’s blood pressure, initiation of angiotensin converting enzyme inhibitors (ACE-I) and/or angiotensin II receptor blockers (ARBs) for renal protection, and control of cholesterol through statin medications [7].

## Conclusions

Renal disease is one of the most serious complications of SLE and affects two-third of lupus patients. Typically, renal failure develops over years of active disease. The patient presented in this case had an extremely rare presentation given her rapidly progressive renal failure. CG is a new clinicopathological entity described in patients with lupus that present with rapidly progressive renal failure. CG is most commonly seen in African American women and is associated with HIV, as reported in an article by Pinto, et al. However, there are a handful of case reports of CG being described in association with autoimmune diseases and SLE. Patients with lupus-related CG have more severe findings at presentation as well as acutely worsening clinical courses similar to the patient described in this case. These patients have a poorer renal prognosis and develop end-stage renal disease (ESRD) within 13 months on average. Lupus nephritis improves in pregnancy if patients are on treatment for SLE. However, in untreated patients, lupus nephritis worsens. The patient presented in this case likely had a degree of underlying glomerulonephritis prior to her pregnancy given her mild proteinuria in the previous ED visits, but pregnancy likely escalated the patient from mild glomerulonephritis to hyperacute renal failure with CG.
